# What Have We Learnt from the Recent Multimodal Managements of Young Patients with ATRT?

**DOI:** 10.3390/cancers17071116

**Published:** 2025-03-26

**Authors:** Sylvia Cheng, Chantel Cacciotti, Carol L. S. Yan, Lucie Lafay-Cousin

**Affiliations:** 1 British Columbia Women and Children’s Hospital, Vancouver, BC V6H 3N1, Canada; 2 London Health Center, London, ON N6A 5W9, Canada; 3 Alberta Children’s Hospital, Calgary, AB T3B 6A8, Canada

**Keywords:** ATRT, high-dose chemotherapy, intrathecal chemotherapy, radiotherapy, maintenance therapy molecular subgroup

## Abstract

Atypical teratoid rhabdoid tumors are rare embryonal tumors of the nervous system mainly seen in very young children with aggressive behavior and dismal prognosis when treated with conventional chemotherapy only. More recent multimodal strategies combining, variably, high dose chemotherapy, radiotherapy and or intrathecal chemotherapy have led to some stride in survival. We present the results of the most recent clinical trials and registry data for patients treated with these multimodal approaches with survival ranging from 37.1% to 88.9%. We review the current consensus of the molecular characterization of these tumors into 3 subgroups (ATRT-TYR, ATRT-SHH and ATRT-MYC) and discuss the potential clinical impact of molecular subgrouping on survival. We explore other therapeutic tools including intrathecal chemotherapy and maintenance and possible new targeted agents for patients failing multimodal strategies

## Simple Summary

Atypical teratoid rhabdoid tumors are rare and aggressive CNS tumors posing a therapeutic dilemma given the early age of onset and the vulnerability of the developing brain to treatment-related toxicity. Most recent treatment protocols have relied on multimodal approaches with modest increments in survival. In this manuscript, we provide an overview of the results of the most recent clinical trials for ATRT and describe our current understanding of the contribution of the molecular characterization of these tumors to inform the next generation of clinical trials for ATRT.

## 1. Introduction/Historical Background

Since its initial histopathological description in the late 1990s, the management of atypical teratoid rhabdoid tumors (ATRTs) has evolved but continues to pose significant challenges, and their prognosis remains unsatisfactory. ATRT is a rare entity of embryonal tumors which accounts for 1 to 2% of all pediatric CNS tumors, with 2/3 of the patients presenting before the age of 36 months, and up to 50% are diagnosed in the first year of life [1,2]. The development of dedicated clinical trials for ATRT has led to improvement in survival compared to dismal historical outcomes but at a significant cost given the very young age of these patients at diagnosis and the increased vulnerability of the developing brain to treatment-related toxicity, notably with radiotherapy (RT). While some studies have suggested survival benefits with adjuvant RT, its role remains controversial [3,4,5].

Earlier experiences using conventional chemotherapy both in European and North American trials for young children with malignant brain tumors resulted in dismal survival rates for patients with ATRT ranging from 0 to 13% [6,7]. The first prospective clinical trial for children with ATRT with intensified chemotherapy using an IRSIII doxorubicin-based regimen, along with serial intrathecal injections of chemotherapy prior to adjuvant RT, led to a 2-year PFS, and OS was 53 ± 13% and 70 ± 10%, respectively. While this survival figure constituted a significant increment in survival compared to historical series, a neurocognitive assessment of the survivors was not embedded in the study to report on the potential impact on the intellectual outcomes of this strategy [8]. Similarly, the European Rhabdoid Registry (EU-RHAB) reported improved 6-year EFS and OS reaching 45 ± 9% and 46 ± 10%, respectively, in patients treated with doxorubicin- and ifosfamide-based regimens in combination with serial intraventricular chemotherapy and focal or craniospinal irradiation [9]. More recently reported, the St Jude SJYC07 trial enrolled 52 patients with ATRT who received conventional induction chemotherapy, including high-dose methotrexate (HD MTX) followed by consolidation with RT based on age and metastatic status and maintenance therapy. The 23 patients with localized disease had a 5-year PFS of 39.1 ± 11.5% and OS of 51.8 ± 12%, while none of the patients with disseminated disease survived [10].

## 2. HDC for Atypical Teratoid Rhabdoid Tumors

Consolidation with high-dose chemotherapy (HDC) and stem cell transplantation has been one of the alternative strategies used to avoid or delay the use of craniospinal irradiation in young children with CNS embryonal tumors. Some of those early trials of HDC for infant malignant brain tumors included ATRT patients. In the phase I/II CCG 99703 trial, eight patients with localized ATRT received three sequential cycles of high-dose carboplatin and thiotepa following three cycles of induction. The reported 5-year EFS for them was 37.5 ± 17.1%, with one of the four survivors never receiving irradiation [11]. Gardner et al. reported the experience of 13 patients with ATRT treated on Headstart I and II (HSI, HSII) trials using one single cycle of high-dose carboplatin, etoposide, and thiotepa. While no survivors were reported on HSI, the 3-year EFS for the seven patients treated on HSII was 43 ± 19%, with none of the three survivors receiving irradiation [12].

In Europe, Benesch et al. reported 19 children from the EU-RHAB registry treated with multimodal therapy, including HDC using various regimens of consolidation but predominantly one cycle of carboplatin and thiotepa. Fourteen patients (73.7%) received RT at some point in their treatment [13]. The estimated 2-year PFS and OS were 29% (±11%) and 50% (±12%), respectively. From this experience, the authors suggested that some patients with ATRT might benefit from HDC with adjuvant RT but called for further evaluation of the impact of this approach in a prospective and randomized manner [13].

The Medical University of Vienna developed an intensive multimodal regimen for patients with ATRT (MUV-ATRT) consisting of induction chemotherapy with doxorubicin, cyclophosphamide, vincristine, ifosfamide, cisplatin, etoposide, and methotrexate augmented with the serial injection of intrathecal therapy (etoposide and depo cytarabine), followed by one cycle of HDC identical to that of the HeadStart consolidation (carboplatin, etoposide, and thiotepa) and focal RT. The reported 5-year EFS and OS for the 13 consecutive patients treated with this regimen were 88.9 ± 10.5% and 100%. The toxicity of this intensive regimen was deemed tolerable with expected myelosuppression, but there was significant ototoxicity with six (46%) patients requiring hearing support. Neurocognitive evaluation was obtained in nine patients, indicating age-normal scoring within five patients [14]. Based on a limited number of patients, this report provided encouraging survival and described the impact on intellectual outcomes in this young group of patients.

COG trial ACNS 0333 was the largest North American prospective clinical trial specifically dedicated to ATRT based on HDC, which enrolled 65 patients. The protocol included two cycles of induction with HD MTX, three cycles of consolidation with high-dose carboplatin and thiotepa, and age-based focal radiotherapy for all patients. Craniospinal irradiation (CSI) was recommended but not mandated for patients with disseminated disease at diagnosis. Twenty-eight patients received adjuvant focal RT, while twelve underwent CSI. The timing of adjuvant RT, either prior to or after consolidation with HDC, was not associated with significant difference in outcome. This HDC-based multimodal approach led to the significant improvement of survival compared to historical controls, bringing up the 4-year EFS and OS to 37% and 43%, respectively, while reporting a toxicity-related mortality of 6%. No statistical difference in survival was identified according to metastatic status or extent of resection. While no direct comparison can be made, the survival figure for patients with localized ATRT was in a similar range to the one reported in the SJYC07 protocol not based on HDC [15].

Although the goal of these multimodal regimen was to minimize the risk of deleterious side effects of craniospinal irradiation in young children, limited series have described long-term survivors completely spared from adjuvant RT. In the Canadian ATRT retrospective registry, the 2-year OS for the 18 patients treated with HDC was 47.9% (±12.1%). Most notably, 11 of these patients never received adjuvant RT, and 6 of 9 survivors (66.6%) treated with HDC did not receive RT [16,17]. A retrospective review of the neuropsychological status was available for 50% of the survivors from the Canadian ATRT registry. All except one were treated with HDC. Three received focal RT, and one underwent adjuvant CSI. Despite the limited use of adjuvant RT, the overall cohort appeared significantly impaired at school age. However, three more recently treated patients had an average to high average FSIQ, potentially suggesting that the improvement of supportive care and surgical technique along with the omission of large-field RT may lead to more preserved intellectual outcomes [18]. These limited experiences indicate that survival without RT is possible; however, the exact characterization of the patients who can safely be spared from RT still needs to be delineated.

**Figure 1:**
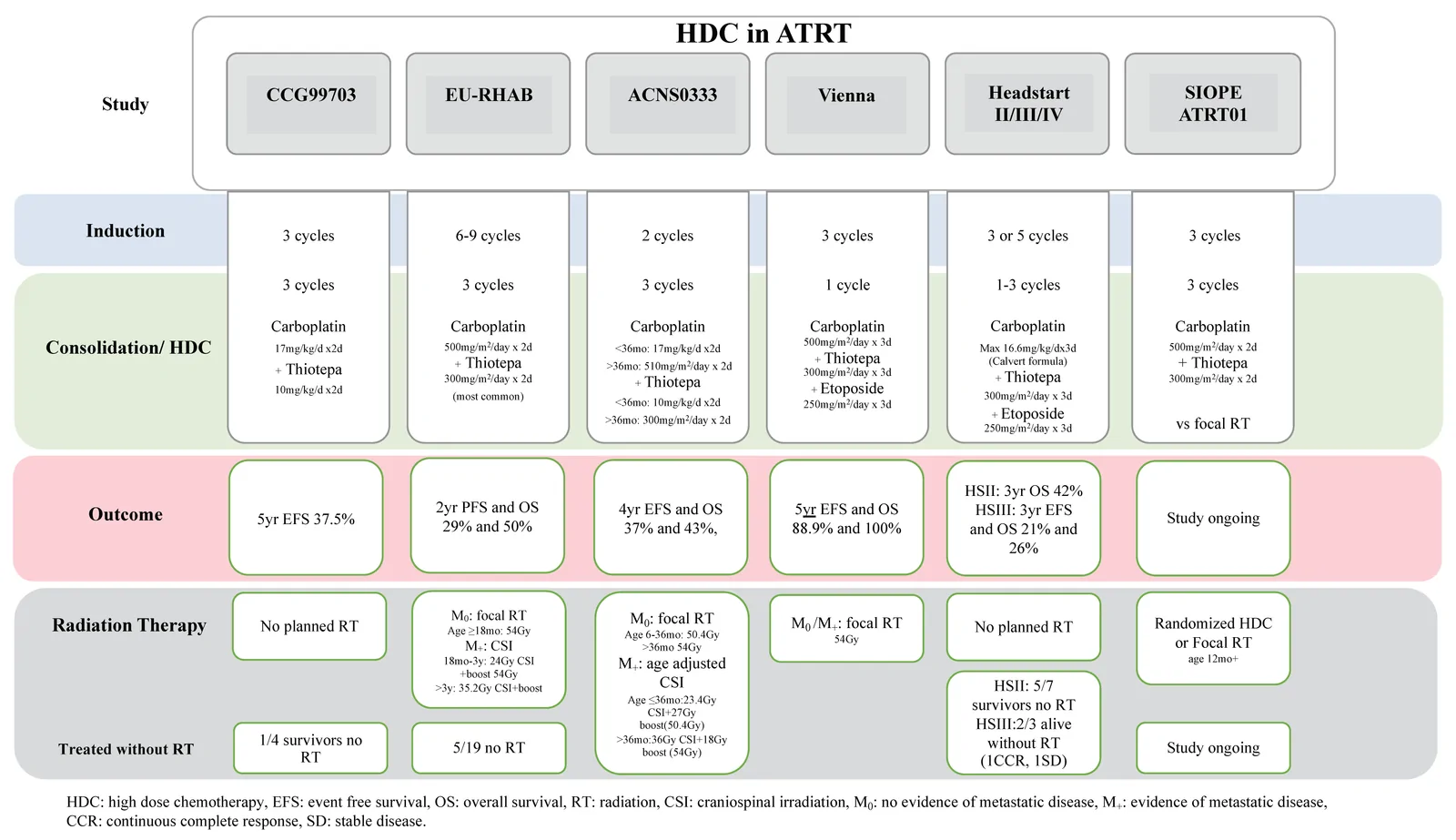
Summary of most recent trials based on the high-dose chemotherapy strategy.

Building on the above-mentioned experiences, the ongoing European prospective trial SIOPEATRT01 is investigating, in a randomized fashion, the impact of three cycles of HDC (carboplatin, thiotepa) in comparison to focal RT as consolidation therapy. The study plans to accrue 152 patients. Following a common induction phase of conventional chemotherapy (doxorubicin, ICE, VCA, and intraventricular methotrexate), patients between 12 months and 35 months of age and localized disease will be randomized between HDC or focal RT for consolidation. Patients less than 12 months will proceed to consolidation with HDC and no RT, while children ≥ 36 months will receive consolidation with RT according to their metastatic status. This trial, therefore, constitutes, to date, the first prospective trial investigating the omission of RT in a defined group of young patients with ATRT (Figure 1).

## 3. Contribution of Molecular Characterization

### 3.1. Molecular Landscape of ATRT

Distinct molecular subgroups have been identified among the ATRTs from various large retrospective cohorts [19,20,21,22]. Collaborative international efforts have aimed to align these findings and led to a pivotal consensus paper [23] defining three molecular subgroups of ATRT, namely ATRT-TYR, ATRT-SHH, and ATRT-MYC, each exhibiting discrete clinical and molecular characteristics.

**Figure 2:**
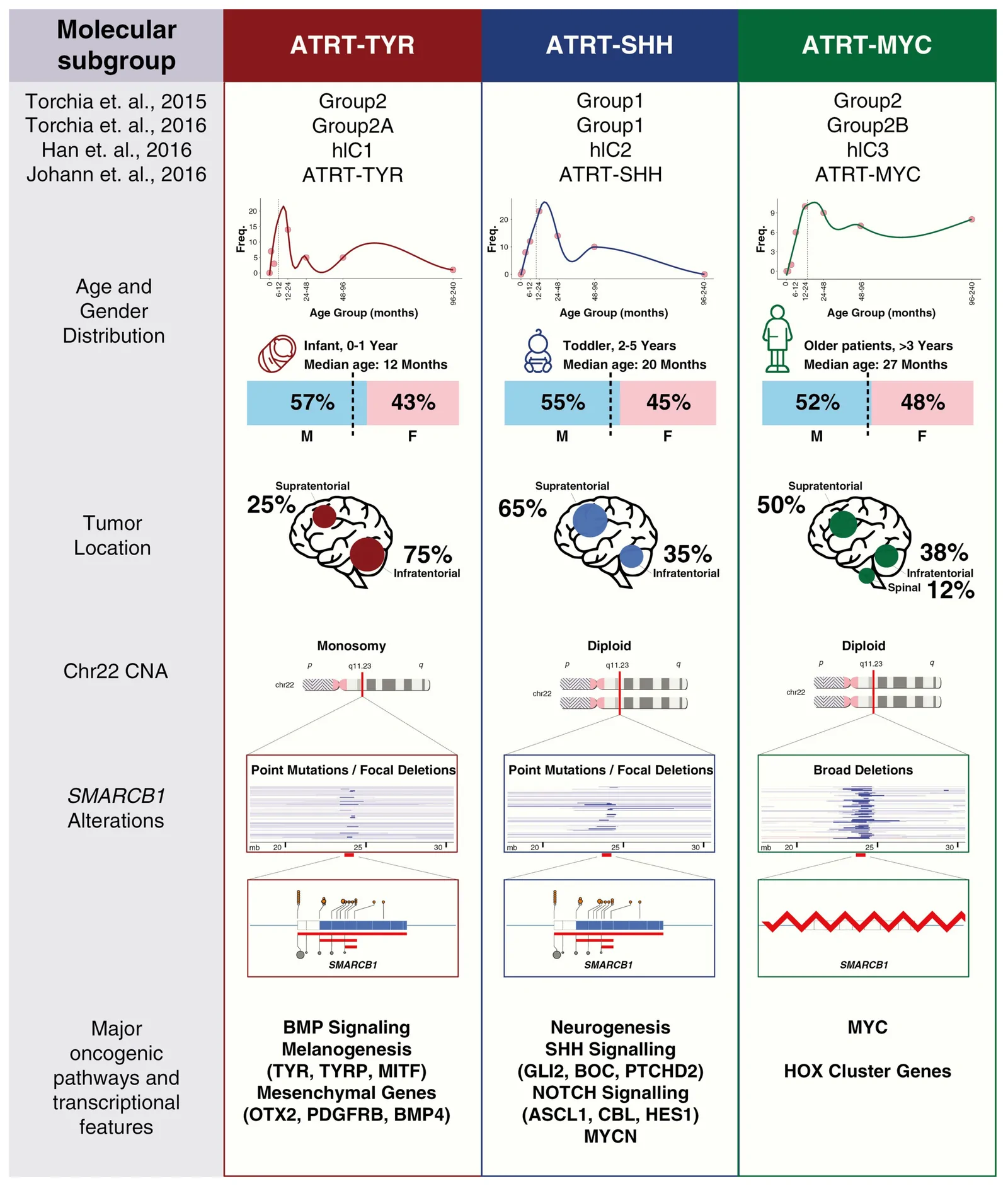
Consensus overview of ATRT molecular subgroups (taken from Ho et al. [19,20,21,22,23]).

The ATRT-TYR subgroup predominantly occurs in younger patients, with a median age of diagnosis at 12 months, whereas ATRT-MYC tumors are more commonly found in older children, with a median age of 27 months. Most ATRT-TYR tumors are infratentorial (75%), while most ATRT-SHH tumors (65%) and 50% of ATRT-MYC tumors arise from the supratentorial region. Notably, all spinal ATRTs are classified within the ATRT-MYC subgroup (12%) (Figure 2).

Further DNA methylation analyses have delineated three subgroups of ATRT-SHH named SHH-1A, 1B, and 2, which may represent determinants of different clinical outcomes or surrogates of alternative driver mutations [24,25]. Unique pathway upregulations are evident in the gene expression profiles across the three subgroups. ATRT-TYR is linked to neuroepithelial lineage, while neuronal genes are overexpressed in ATRT-SHH, and mesenchymal genes are predominant in ATRT-MYC. Interestingly, ATRT-TYR and ATRT-MYC also exhibit overlapping gene sets related to immune response [23]. ATRT-MYC tumors share genetic and epigenetic similarities with extracranial rhabdoid tumors, displaying both comparable DNA methylation profiles and global hypomethylation. Studies have indicated increased cytotoxic T-cell infiltration and elevated expression of immune checkpoint regulators (PD-1 and PD-L1) in these tumors [26]. Genome-wide hypermethylation has been detected in both the ATRT-TYR and ATRT-SHH groups [21]. Some of these alterations may represent future actionable therapeutic targets specific to subgroups. Lastly, ATRTs are, for the large majority, SMARCB1-deficient tumors, but in rare cases, they can be associated with the mutation of SMARCA4 (0.5–2% of all ATRTs) [21,27]. These tumors are associated with a younger age at diagnosis (median, 3 months), inherited germline mutations (in 73% of cases), and a poorer prognosis [28,29].

### 3.2. Prognostic Value of ATRT Molecular Subgroups

While we have gained significant understanding of the molecular landscape of ATRT, the evaluations of the prognostic value of the three molecular subgroups from the most recent registry series or prospective clinical trials remain conflicting.

In COG trial ACNS 0333, the ATRT-SHH, ATRT-TYR, and ATRT-MYC subgroups accounted for 30%, 43%, and 27% of patients, and in the post hoc exploratory analysis, the 4-year OSs associated with SHH, TYR, and MYC subgroup were, respectively, 56%, 41%, and 27%. The sample sizes of each group were too small to detect significant differences [15], but the survival pattern was aligned with previous retrospective reports, whereby ATRT-SHH may represent a group more responsive to therapy.

Conversely, in the EU-RHAB registry, non-TYR subgroups and age under 1 year were identified as independent risk factors. Patients presenting with both risk factors exhibited significantly worse outcomes (5-year OS of 0%), in contrast to those without these risk factors (i.e., ATRT-TYR and age ≥ 1 year), who had a 5-year OS of 71.5% [27].

The evaluation of the prognostic value of molecular subgrouping in the cohort of ATRT from the St Jude trial SJYC07 described a more favorable prognosis for the ATRT-TYR subgroup in children under 3 years (5-year OS of 58.8 ± 11.9%). Patients in the ATRT -TYR subgroup were more likely to be non-metastatic at diagnosis. The comparison by methylation subgroup in non-metastatic patients did not indicate significant differences in outcome. In their experience, ATRT-TYR tumors typically presented as localized posterior fossa tumors and demonstrated indolent progression with longer survival compared to other subgroups. A less favorable outcome for the ATRT-SHH subgroup was also associated with more frequent metastatic disease [10]. Analyzing the SJCY07 and two other clinical trial cohorts, Tran et al. found no significant difference in patient survival among the subgroups of ATRT-SHH (SHH-1A, SHH-1B, and SHH-2) in frontline clinical trials to support using them in risk stratification or clinical decision making [30].

It is essential to recognize that treatment strategies varied across studies. HDC was a key component of multimodal treatment in ACNS0333, whereas in the EU-RHAB registry, it was administered in only 24% of patients, and in the St Jude trials, non-myeloablative HDC was exclusively used in older children (>3 years). The EU-RHAB regimen was an anthracycline-based chemotherapy in combination with serial intrathecal chemotherapy, while children treated in the SJYC07 trial underwent maintenance oral chemotherapy. The indication and modalities of adjuvant RT, although mostly driven by age and risk group, also varied from one protocol to another. Therefore, the interplay between treatment intensities and molecular subgroups must be considered when evaluating the individual prognostic significance of each molecular subgroup.

## 4. Who Benefits from These Intensive Multimodal Therapies?

The most recent clinical trials using multimodal therapy have led to moderate strides in survival but have not permitted the delineation of the respective contribution of each modality to improved survival. Furthermore, within strategies relying on intensification with HDC, the event-free survivals reported range widely from 29% to 88.9%. Of interest, patients treated with the third HeadStart protocol (HSIII), using one cycle of HDC consolidation after five cycles of induction, had worse outcomes compared to those treated on the previous HSI and II using the same HDC consolidation administered after one to five cycles of induction, and had a median time to progression of 4.1 months, possibly suggesting that the earlier timing of consolidation with HDC is important [12,31].

These data also indicate that despite significant intensification, a sizeable proportion of patients do not benefit from HDC-based multimodal approaches, as suggested by the poor overall survival of 26.7% for the MYC group in the ACNS0333 trial, which remains close to the historical control survival rates and for whom new therapeutic strategies are urgently needed. The survival rates reported in the SJYC07 trial for the patients with localized disease (M0) treated with conventional chemotherapy, focal RT, and maintenance (5-year PFS of 39.1 ± 11.5%) raises the question of the need for intensification with HDC for this clinical subgroup, especially for the ATRT-TYR subgroup (5-year OS of 58.8 ± 11.9%), given the similar outcome reported with the HDC strategy used in ACNS0333 (4-year OS of 41% 95% CI 22–60) [10,15].

It would be of great interest to identify, through molecular and clinical characterization, patients expected to respond favorably to the HDC strategy without adjuvant RT. In their initial integrated genomic and clinicopathological analysis of 259 patients with ATRT, Torchia et al. reported the OS of 16 patients with group 1 ATRT (ATRT-SHH) treated with HDC and no neuroaxis irradiation, estimated at 34% (95% CI 7–61), providing some insight on the molecular profile on those patients who may respond to HDC only. However, the number of patients remained too small in this retrospective cohort to derive any clinical decision making [20]. The ongoing SIOP ATRT 01 trial, which includes a treatment arm with HDC and no planned adjuvant RT for all patients under 12 months of age and in a randomized manner for the patients 12 to 35 months, will explore the relationship between molecular subgrouping and the clinical characteristics of the patients and may provide additional insight on this group of patients.

## 5. Approach to Relapse/Recurrent ATRT

The risk of relapse with any given treatment strategy is highest within 2 years from diagnosis, with most patients relapsing within the first year [15]. Prognosis remains dismal at the time of disease relapse, and salvage treatments vary with no standard approach [32]. Furthermore, published data on salvage therapies options are scant. The St. Jude single-institution trial with 64 children with recurrent/progressive ATRT demonstrated grim outcomes with only five (7.8%) patients alive at median follow-up of 10.9 years (range: 4.2–18.1 years) from progression [32]. The 2-year PFS post relapse and OS were 3.1% (±1.8%) and 1.6% (±1.1%), respectively. In univariate analysis, older age at diagnosis, female gender, ATRT-TYR group, and a metastatic site of progressive disease compared to local or combined sites were associated with longer survival. Children within the ATRT-TYR group (n = 10) had a better post-relapse OS compared to those with ATRT-MYC (n = 11) (2-year survival estimates: 60.0 ± 14.3% vs. 18.2 ± 9.5%; *p* = 0.019), or those with ATRT-SHH (n = 21; 4.8 ± 3.3%; *p* = 0.014). Twenty-five (39%) patients underwent palliative therapy with no further tumor directed treatment, and all died shortly after. Salvage therapies were variable and included chemotherapy or molecular targeted therapy only in 27% (n = 17), a combination of chemotherapy, molecular targeted therapy, and RT in 31% (n = 20), and RT alone in 3% (n = 2). The 2-year post-relapse OS of patients who received any salvage therapy was 33.3 ± 7.3%. Although this may suggest some benefits in prolonging survival in a limited number of patients, relapse ATRT rarely appeared salvageable [33].

Radiation therapy is often considered in patients with relapsed disease, especially if not used in the upfront treatment [3]. Within the St. Jude cohort of relapse ATRT, CSI was employed in 23% of patients who received salvage therapy with curative intent. Two of these nine patients were long-term survivors. The median time to death from CSI was 21.6 months (range: 0.4–37.0 months), suggesting again a potential role for life prolongation for CSI [32]. But the use of CSI remains controversial, even in the relapse setting, given the young age of these patients, which has led some groups to explore alternative administration means for chemotherapy, such as metronomic chemotherapy regimens with anti-angiogenic purposes. The MEMMAT regimen, using multiagent anti-angiogenic drugs and chemotherapy combined with serial injections of intrathecal chemotherapy, has been investigated in recurrent CNS embryonal tumors, including ATRTs. Peyrl et al. reported three patients with relapse ATRT, one with multiple recurrences, treated with this regimen [33]. All three patients were reported alive at 42, 12, and 10 months post last recurrence with one demonstrating partial response, one stable disease, and the third no disease after surgical intervention [33].

Outside of the salvage RT or conventional chemotherapy, limited reports are available on the use and possible effect of targeted agents in relapse ATRT. Tazemetostat, an EZH2 inhibitor, currently remains the most tested single agent. Tazemetostat was initially investigated for adults or pediatric patients with deficient SMARCB1 or SMARCA4 tumors of diverse types, indicating some objective responses. The Children’s Oncology Group Pediatric MATCH trial (APEC1621C or NCT03213665) was a phase II trial evaluating tazemetostat in pediatric patients with recurrent/refractory tumors harboring EZH2 mutations or loss of SMARCB1 or SMARCA4. Of the 20 patients included, 8 had ATRT, and only 1 of them demonstrated disease stabilization [34]. Overall, the 6-month PFS and OS were 35% and 45%, respectively. The investigators concluded that although tazemetostat did not meet the primary efficacy endpoint in this population of refractory pediatric tumors, 25% of patients with multiple histologic diagnoses experienced prolonged stable disease for 6 months and over (range = 9–26 cycles), suggesting a potential effect of tazemetostat on disease stabilization as reported by other groups [35,36].

A phase II study (SJATRT) using an Aurora kinase A inhibitor, alisertib, in patients < 22 years with recurrent ATRT demonstrated the tolerability of single-agent alisertib, with about one third of patients experiencing stable disease or partial response. Amongst the 30 patients, eight patients had stable disease, and one a partial response leading to a projected PFS of 30% at 6 months and 13.3% at 12 months. The OS was 36.7% in this relapsed cohort, suggesting the Aurora kinase inhibitor as an alternative option for ATRT recurrence [37].

Ongoing early-phase clinical trials with immunotherapy agents may be available for patients with relapsed ATRT. The combination of a PD-L1 (Atezolizumab) with a TGIT-targeted antibody (Tiragolumab) is currently under investigation in a phase I/II study for patients with SMARCB1- or SMARCA4-deficient tumors, including those with ATRT (NCT05286801 or PEPN2121).

Overall, the expected survival of relapsed ATRT is minimal, stressing the need to optimize upfront therapy for these aggressive and rapidly progressing tumors. As we have likely reached the maximum intensity of treatment that HDC can offer, especially in combination with other modalities like RT, other modalities of drug administrations to enhance brain tumor penetration may represent additional tools for the therapeutic arsenal in ATRT.

## 6. Future Perspectives and Research

### 6.1. Exploring the Role of Intrathecal Therapy (It) in Upfront Treatment for ATRT

In children with acute lymphoblastic leukemia (ALL), the substitution of intrathecal chemotherapy for brain irradiation in the prophylactic treatment of the CNS in combination with systemic chemotherapy has allowed increased drug penetration to the neuroaxis and resulted in fewer long-term treatment-related sequelae, including fewer second primary tumors [38] and less neurocognitive impairment. It has become an essential component of the treatment backbone for pediatric acute leukemia for decades [39].

This modality of drug administration is of particular interest for young patients with CNS tumors, which are at higher risk of treatment-related toxicity in the developing brain. Furthermore, as 25–30% of children with ATRT present with CNS spreading at initial diagnosis and 35 to 40% will present with metastatic relapse, this underscores the gaps in CNS prophylaxis and in targeting residual microscopic disease [9,17,27,32].

The German group HIT SKK introduced intraventricular or intrathecal (IT) chemotherapy in their first-generation protocol for the medulloblastoma of early childhood in the early 1990s as an alternative to adjuvant craniospinal irradiation. While describing very encouraging survival in a subset of patients with nodular desmoplastic medulloblastoma, their report also indicated a less pronounced neurocognitive deficit when compared to historical cohorts treated with RT [40]. The combination of CNS prophylaxis with IT with systemic conventional chemotherapy remains the backbone of their protocol for young children with CNS embryonal tumors. There are limited intrathecal agents available and tested in children, and the use of IT chemotherapy specifically in pediatric ATRT is limited, but there has been extensive experience using IT therapy to young children with pediatric CNS embryonal tumors to establish at least its feasibility and safety (Supplemental Table S1). The first prospective trial for ATRT from the DFCI, based on IRS conventional chemotherapy and adjuvant RT, also included serial methotrexate-based IT [8]. As described above, patients treated with the HDC regimen in the EU-RHAB registry or patients treated with the MUV-ATRT protocol at the University of Vienna also received serial injections of IT as part of their initial therapy [13,14]. The Hospital for Sick Children piloted an RT-sparing regimen between 2005 and 2023 for ATRT patients with IT Topotecan and cytarabine for CNS prophylaxis and/or treatment, and a 12-month maintenance regimen added to the CCG99703 HDC backbone. IT Topotecan was selected based on phase II studies of metastatic CNS embryonal tumors [11,41] and their own institutional experience. To counter drug resistance, IT cytarabine was used during induction, and IT Topotecan was used during maintenance. Grade 1–2 arachnoiditis and pseudo-tumor cerebri were observed in 10% and 5% of patients, respectively, during maintenance, but overall, they were well tolerated [42].

In terms of impact on survival, a meta-analysis of observational studies indicated that patients who received IT chemotherapy as part of their treatment had significantly longer OS than those who did not. The survival for the 30 treated with IT chemotherapy was significantly higher with a 2-year OS of 64% (95% CI, 46.5–82.0) compared to 17.3% (95% CI 5.4–29.3; *p* < 0.0001) along with a low prevalence of distant CNS metastasis compared with those without IT therapy (n = 49) (20% vs. 59.2%; *p* = 0.001). In children ≤ 3 years, the mean OS (n = 20) was 16.1 months for those who received IT vs. 11.7 months in those without IT n = 30) [43] but there was no significant difference in OS for the group of patients older than 3 years (*p* = 0.55). However, the authors mentioned that almost all older patients also underwent radiotherapy. Given the safety and promise of additive efficacy reported, this modality of chemotherapy administration may represent an opportunity to address the therapeutic gap without the added toxicity of RT or cytotoxic agents in these young ATRT patients. The Canadian Pediatric Brain Tumor Consortium is currently developing a national pilot study to assess the feasibility of combining serial intrathecal/intraventricular therapy to an HDC backbone and maintenance therapy without adjuvant RT. Nevertheless, although the aim of intrathecal chemotherapy for the prophylaxis or treatment of neuro axis dissemination is to avoid the negative neurocognitive impact of CSI in young patients, we are still lacking robust neuropsychological studies to describe the specific impact and long-term effect of repeated IT injections. Furthermore, in most of the recent multimodal approaches, the associated use of RT, even focal, remains a confounding factor for evaluating the individual impact of IT chemotherapy on neurocognitive outcomes. Future studies exploring the impact of IT on ATRT should therefore investigate the neurocognitive status of these patients.

### 6.2. Exploring the Role of Maintenance Therapy in ATRT

The short period of time to the relapse of ATRT and other young patients with CNS embryonal tumors following the end of treatment, as well as the failure of complete remission to predict a lower rate of relapse, has led to the investigation of differentiation agents, such as differentiating agents and other metronomic therapies for a “maintenance” regimen in younger children with ATRT. In the St Jude SJYC07 trial, all patients underwent 6 months of maintenance chemotherapy, including cyclophosphamide, topotecan, and erlotinib after consolidation with RT. Progressive disease (PD) was observed largely during the pre-maintenance phase of the treatment. For the five patients who completed consolidation without progression, no PD was detected during the maintenance. The median time for the completion of maintenance therapy for PD for those who subsequently failed was 12.7 months (0–67.2 months) (10). The early progression of therapy prior to maintenance in most of the patients did not provide any indication of the potential benefit of the addition of maintenance in this series.

Based on cell culture studies indicating that ATRT cells exhibit relatively undifferentiated cell phenotypes with elevated levels of CCND1 and in vitro studies showing fenretinide, a synthetic retinoid, and 4-OH-tamoxifen (4OHT) synergistically down modulating CCDN1 and inducing apoptosis in ATRT cells [44,45], the combination of Isotretinoin (cis-retinoic acid, Accutane©, RA) and 4OHT (tamoxifen) has been used as maintenance therapy post HDC consolidation in the Hospital for Sick Children’s institutional standard of care protocol for CNS embryonal tumors for young children with good tolerance and minimal toxicity (Annie Huang personal communication). The national pilot study from the Canadian Pediatric Brain Tumor Consortium plans to incorporate the combination of isotretinoin and 4OHT for maintenance in the low-risk arm of their protocol.

### 6.3. Preclinical Drug Screening for Molecularly Informed Treatment

While the prognostic value of molecular subgrouping remains conflicting based on the data of the most recent prospective trials and registries, preclinical investigations, such as drug screening in specific molecular subgroup models, may introduce new therapeutic perspectives to test in future clinical trials. Torchia et al. performed a limited drug screen of agents targeting subgroup-specific pathways in ATRT-SHH and ATRT-MYC cell lines [19,20]. This initial drug screening indicated therapeutic vulnerabilities specific to different tumor subgroups. ATRT-SHH tumors seem to rely more heavily on various targetable epigenetic regulators for their survival compared to other ATRT subtypes. Although the use of EZH2 inhibitors might be more favorable in this group, the molecular characterization of the patients treated in early phase I and II with tazemetostat was not available to evaluate those preclinical signals. While ATRT-SHH tumors have been reported to present with overexpression of the SHH pathway genes (GLI2, BOC, PTCHD2, MYCN) [21], it is thought that SHH signaling in ATRT-SHH is likely an indirect consequence of SMARCB1 loss, rather than the primary molecular driver of these tumors [23], limiting the rational to introduce SHH inhibitor for this subgroup [23]. Unlike ATRT-SHH tumors, the ATRT-MYC and ATRT-TYR subtypes are notably dependent on receptor tyrosine kinase (RTK) signaling, especially the PDGFR pathway. Tyrosine kinase inhibitors (TKIs) such as dasatinib and nilotinib show selective toxicity against ATRT-MYC cell lines, with dasatinib significantly enhancing survival in an intracranial orthotopic xenograft model [19,46]. In addition to small molecule inhibitors targeting specific molecular subgroups, T cell-based immunotherapy is gaining recognition as a potentially effective treatment for rhabdoid tumors. In an ATRT-MYC syngeneic mouse model, blocking PD-L1 led to a significant reduction in tumor growth and improved survival for the tumor-bearing mice [47]. Theruvath et al. found that ATRTs, unlike normal infant or pediatric brain tissue, express B7-H3, a target currently being explored in clinical trials for immunotherapy [48]. In a patient-derived xenograft model, the injection of B7-H3-targeting chimeric-antigen receptor (CAR) T cells, either intratumorally or intraventricularly, resulted in tumor regression in all tested animals [48]. Together, these findings suggest that T cell-based immunotherapy could be a promising therapeutic approach for rhabdoid tumors, including ATRT, particularly ATRT-MYC. Currently, B7-H3 CAR-T cell clinical trials for children with relapsed/refractory solid tumors are open at several sites (NCT04897321, NCT04185038) [49].

## 7. Conclusions

The most recent multimodal strategies have moderately improved survival in ATRT but still in an unsatisfactory manner. Despite collaboration through multicenter studies and dedicated clinical trials for patients with ATRT, several questions remain unanswered. The clear delineation of patients benefiting from these aggressive strategies is still to be uncovered. Similarly, the respective role of each of the combined modalities needs to be investigated further. The de-escalation of therapy is clearly not the goal of the upcoming clinical trials for ATRT, but the ongoing European trial ATRT01 will help to delineate more specifically the role of high-dose chemotherapy and radiotherapy.

The identification of various molecular subtypes in ATRT has not led yet to integrated molecular and clinical risk prognostication, and the prognostic value of these molecular subgroups should be investigated prospectively in the next generation of clinical trials. A meta-analysis of molecularly characterized patients treated with recent multimodal approaches may offer further insight into risk stratification. This could be achieved through international collaboration creating common databases for ATRT patients

Deeper understanding of the underlying molecular pathways involved in specific ATRT subgroups may pave the way toward new therapeutic options for these aggressive tumors. Through further evaluation in preclinical and clinical settings of the underlying biology of these tumors, targeted agents and immune-directed therapies may be added to the therapeutic arsenal for these patients.

While aiming at further advancing the cure rate of ATRT, it is crucial to keep in mind the very young age of these patients to develop new therapeutic strategies preserving neurocognitive outcomes.

## Data Availability

The original contributions presented in this study are included in the article/Supplementary Material. Further inquiries can be directed to the corresponding author.

## Supplementary Materials

The following supporting information can be downloaded at: https://www.mdpi.com/article/10.3390/cancers17071116/s1, Table S1: Intrathecal Chemotherapy Use in Medulloblastoma and other CNS embryonal tumors. Refs. [40,50,51,52,53,54,55,56,57,58] are cited in Table S1.
