# Supplementary material for: What Have We Learnt from the Recent Multimodal Managements of Young Patients with ATRT?
Source: Cancers (Basel). 2025 Mar 26;17(7):1116. doi: 10.3390/cancers17071116 (PMC11987908; doi:10.3390/cancers17071116)
Supplement: Supplementary file 1 [file cancers-17-01116-s001.zip › cancers-3483251-supplementary.pdf]

Intrathecal Chemotherapy Use in Medulloblastoma and other CNS embryonal tumors

Study	Disease	IT Therapy	Other Concurrent Chemotherapy	Toxicities	Leukoencephalopathy on MRI$	Neurocognitive Outcomes	5yr EFS/OS (%)
Rutkowski S et al, NEJM 2005(1)<br>**(HIT-SKK1992)**	Medulloblastoma<br>N=43 (23 classical histology, 20 desmoplastic)<br><br>All infants <3 years	**Intraventricular MTX**<br>2mg/day (36 doses)#<br>Week 1 day 1-4<br>Week 3 day 1-2*<br>Week 5 day 1-2*<br>Week 7 day 1-4<br>(max=72mg)	Cyclo<br>Vincristine<br>MTX 5g/m2<br>Carboplatin<br>Etoposide	-Cerebral seizures(2)<br>-CSF bacteremia (1)<br>-Abdominal ascites with VP shunt (2)	-None (4)<br>-Mild (4)<br>-Moderate (9)<br>-Severe (6)	T scores for all applied tests best in systemic therapy only > intraventricular MTX > Radiation	
Pompe RS, et al, EJC 2015(2)<br>**(HIT 2000)**	Medulloblastoma<br>N=216 received intraventricular MTX<br><br>Age 0-21 years	**Intraventricular MTX**<br>Up to 72mg<br><br>M0 72mg<br>M+ <4y 16-36mg<br>M+ older 48mg	Before 2005, with HDC<br><br>After 2005 metastatic group had CSI	-Reservoir issues (57)<br>-Neurotoxicity (9-including seizures, amnestic aphasia, weakness, termor, headache, nausea, nystagmus)<br>-Infection (21)<br>-Intraventricular hemorrhage (1)<br>-Parenchymal damage (1)<br>-CSF leakage (11)<br>-Death (1 – periventricular disseminated necrotic leukoencephalopathy)	Not evaluated	Not evaluated	57/80
Blaney S, et al, JCO 2005(3)<br>(PBTC-001)<br>Dose finding	CNS embryonal tumors<br>Age <3 years<br><br>N=25	**Intrathecal and/or intraventricular mafosfamide**	18 doses over 17 weeks<br><br>MTD=14mg	Irritability<br>Headache/pain<br>Nausea/vomting<br>Skin discoloration/rash<br>Flusing<br>Anorexia<br>Stridor<br>Arachnoditis<br>Seizure<br>Fever	Not evaluated	Not evaluated	
Blaney S, et al, J Neurooncol 2012(4)<br>(PBTC-001)<br>Pilot	CNS embryonal tumors<br>Age <3 years<br><br>N=71	**Intrathecal and/or intraventricular mafosfamide**	18 doses over 17 weeks	Apnea<br>Arachnoditis<br>Irritability/pain with infusion<br>Cerebral edema<br>Cranial nerve palsy<br>Dehydration<br>Decreased LOC<br>Fever<br>Hallucination<br>Headache<br>Hypertension<br>Hypothermia<br>Hypoxia<br>Increased CSF protein<br>Nausea/vomiting	Not evaluated	Not evaluated	33/51

				Skin discoloration'/rash<br>Seizure<br>Speech impairment<br>Voice changes<br>Weight loss			
Benesch M, et al, J Pediatric Hematol Oncol 2007(5)	Neoplastic meningitis<br>N=5, 1 MB patient	**Intrathecal liposomal cytarabine**	CSI/TBI<br>Various chemo	Transient encephalopathy<br>Seizures<br>Cauda equina syndrome	Not evaluated	Not evaluated	
Yoshimura J, et al, Childs Nerv Syst, 2008(6)<br>(Japan)	Medulloblastoma with leptomingeal disease	**Intrathecal or Intraventricular MTX, ACNU/MCNU**	PE or ICE<br>Radiation	Paraplegia<br>Incontinence<br>Truncal ataxia<br>(attribute all to nitrosoureas)	Not evaluated	Not evaluated	OS 46.4
Clayton J, et al, Acta Neurochir, 2008(7)<br>(Case Report)	Metastatic Medulloblastoma	**Intrathecal topotecan**		Chemical arachnoditis	Not evaluated	Not evaluated	Disease progressed
Chi SN, et al, JCO 2009<br>(DFCI 02-294)	ATRT	**Intrathecal and/or intraventricular MTX, Cytarabine, Hydrocortisone**<br>PO Leucovorin after MTX<br>M0 11 doses<br>M1 weekly until two neg	IRS-III regimen	Not specified	Not evaluated	Not evaluated	2yr PFS/OS<br>53/70
Mastronuzzi A, et al, Anticancer Research 2013(8)<br>(Italy)	Relapsed medulloblastoma (CSF+/leptomeningeal disease)	**Liposomal cytarabine**<br>Q15 days 2mg/kg/dose with PO Dex 5 days	Cisplatin<br>Lomustine<br>Vincristine<br><br>Sorafenib (1)	Hematologic (1)	Not evaluated	Not evaluated	
Yamasaki K, et al, PBC 2019(9)<br>(Japan Registry)	Medulloblastoma<br><br>Infants age <3y (n=17) and Older kids not M0 (n=28)	**Intrathecal MTX**<br><br>6 doses (qmonthly except 1st cycle has 2)	Cisplatin<br>Etoposide<br>Cyclo<br>Vinristine<br><br>HDC<br>Thiotepa<br>Melphalan	No leukoencephalopathy identified			Infants 52.9/51.8<br>(2 local RT, 3 CSI)<br><br>Older kids 82.1/85.7<br>(all had CSI)
Okada K, et al, PBC 2020(10)<br>(Japan PBTC)	Medulloblastoma<br>N=48	**Intrathecal MTX**<br><br>6 doses (qmonthly except 1st cycle has 2)	Cisplatin<br>Etoposide<br>Cyclo<br>Vinristine<br><br>HDC<br>Thiotepa<br>Melphalan<br><br>Radiation	No leukoencephalopathy identified	Not evaluated	Not evaluated	3yr PFS/OS<br>90.5/93.9

*With high dose methotrexate 5g/m2 IV over 24 hours

#31 patients received cumulative doses of >/= 48mg

$T2 MRI mild=spotted, circumscribed lesions; moderate=patchy lesions; severe=confluent lesions

References

1. Rutkowski S, Bode U, Deinlein F, Ottensmeier H, Warmuth-Metz M, Soerensen N, et al. Treatment of early childhood medulloblastoma by postoperative chemotherapy alone. N Engl J Med. 2005;352(10):978-86.
2. Pompe RS, von Bueren AO, Mynarek M, von Hoff K, Friedrich C, Kwiecien R, et al. Intraventricular methotrexate as part of primary therapy for children with infant and/or metastatic medulloblastoma: Feasibility, acute toxicity and evidence for efficacy. Eur J Cancer. 2015;51(17):2634-42.
3. Blaney SM, Boyett J, Friedman H, Gajjar A, Geyer R, Horowtiz M, et al. Phase I clinical trial of mafosfamide in infants and children aged 3 years or younger with newly diagnosed embryonal tumors: a pediatric brain tumor consortium study (PBTC-001). J Clin Oncol. 2005;23(3):525-31.
4. Blaney SM, Kocak M, Gajjar A, Chintagumpala M, Merchant T, Kieran M, et al. Pilot study of systemic and intrathecal mafosfamide followed by conformal radiation for infants with intracranial central nervous system tumors: a pediatric brain tumor consortium study (PBTC-001). J Neurooncol. 2012;109(3):565-71.
5. Benesch M, Sovinz P, Krammer B, Lackner H, Mann G, Schwinger W, et al. Feasibility and toxicity of intrathecal liposomal cytarabine in 5 children and young adults with refractory neoplastic meningitis. J Pediatr Hematol Oncol. 2007;29(4):222-6.
6. Yoshimura J, Nishiyama K, Mori H, Takahashi H, Fujii Y. Intrathecal chemotherapy for refractory disseminated medulloblastoma. Childs Nerv Syst. 2008;24(5):581-5.
7. Clayton J, Vloeberghs M, Jaspan T, Walker D, MacArthur D, Grundy R. Intrathecal chemotherapy delivered by a lumbar-thecal catheter in metastatic medulloblastoma: a case illustration. Acta Neurochir (Wien). 2008;150(7):709-12.
8. Mastronuzzi A, Del Bufalo F, Iacono A, Secco DE, Serra A, Colafati GS, et al. Intrathecal liposomal cytarabine and leptomeningeal medulloblastoma relapse: a valuable therapeutic option. Anticancer Res. 2013;33(8):3515-8.
9. Yamasaki K, Okada K, Soejima T, Sakamoto H, Hara J. Strategy to minimize radiation burden in infants and high-risk medulloblastoma using intrathecal methotrexate and high-dose chemotherapy: A prospective registry study in Japan. Pediatr Blood Cancer. 2020;67(1):e28012.
10. Okada K, Soejima T, Sakamoto H, Hirato J, Hara J. Phase II study of reduced-dose craniospinal irradiation and combination chemotherapy for children with newly diagnosed medulloblastoma: A report from the Japanese Pediatric Brain Tumor Consortium. Pediatr Blood Cancer. 2020;67(11):e28572.
